# The Paucity of Frugivores in Madagascar May Not Be Due to Unpredictable Temperatures or Fruit Resources

**DOI:** 10.1371/journal.pone.0168943

**Published:** 2017-01-13

**Authors:** Sarah Federman, Miranda Sinnott-Armstrong, Andrea L. Baden, Colin A. Chapman, Douglas C. Daly, Alison R. Richard, Kim Valenta, Michael J. Donoghue

**Affiliations:** 1 Department of Ecology and Evolutionary Biology, Yale University, New Haven, Connecticut, United States of America; 2 Department of Anthropology, Hunter College of the City University of New York, New York, New York, United States of America; 3 Departments of Anthropology & Biology, The Graduate Center of the City University of New York, New York, New York, United States of America; 4 The New York Consortium in Evolutionary Primatology (NYCEP), New York, New York, United States of America; 5 Department of Anthropology and McGill School of Environment, McGill University, Montréal, Québec, Canada; 6 Wildlife Conservation Society, Bronx, New York, United States of America; 7 New York Botanical Garden, Institute of Systematic Botany, Bronx, New York, United States of America; 8 Department of Anthropology, Yale University, New Haven, Connecticut, United States of America; Centre for Cellular and Molecular Biology, INDIA

## Abstract

The evolution of ecological idiosyncrasies in Madagascar has often been attributed to selective pressures stemming from extreme unpredictability in climate and resource availability compared to other tropical areas. With the exception of rainfall, few studies have investigated these assumptions. To assess the hypothesis that Madagascar’s paucity of frugivores is due to unreliability in fruiting resources, we use statistical modeling to analyze phenology datasets and their environmental correlates from two tropical wet forests, the Réserve Naturelle Intégrale Betampona in Madagascar, and Kibale National Park in Uganda. At each site we found that temperature is a good environmental predictor of fruit availability. We found no evidence of a significant difference in the predictability of fruit availability between the two sites, although the shorter duration of phenological monitoring at Betampona (two years, versus 15 years at Kibale) limits our ability to infer long-term patterns. Comparisons of long-term temperature data from each site (15 years from Kibale and 14 from Betampona) indicate that temperature is more predictable at Betampona than at Kibale. However, there does appear to be a difference between the two sites in the total fruit availability at any given time, with fruit being generally less abundant at Betampona. Our results appear contrary to the prevailing hypothesis of a selective force imposed by unpredictable resource availability or temperature, and we suggest other possible explanations for Madagascar’s unique biota.

## Introduction

Long isolation and *in situ* diversification in Madagascar have resulted in high levels of endemism, elevated phylogenetic diversity in some taxa, and unique species assemblages characterized by idiosyncratic traits and ecological strategies [1]. These characteristics have been explained in part by extreme seasonality and unpredictable climates on the island as compared with other tropical areas [2–7]. As a prime example, Madagascar is noteworthy for its relative paucity of frugivores—here defined as organisms whose diets consist of 50% or more of fruit by volume [8]–and the fact that the majority of these frugivores are primates [2, 9]. The Energy Frugality Hypothesis (EFH) proposes that Madagascar has an unpredictable climate, causing unreliability in fruiting patterns, and that this has in turn resulted in few obligate frugivores owing to selection against dependence on unreliable resources [6, 7]. But, is it really true that Madagascar is especially unpredictable with respect to climate and resource availability?

Dewar and Richard [2] demonstrated that Madagascar is ‘hypervariable’ in terms of rainfall as compared with other tropical areas, and, furthermore, that this hypervariability differs between the dry west and southwest forests (with greater inter-annual unpredictability) and the wet eastern forests (with greater intra-annual variability). To our knowledge, no other climatic variables in Madagascar have been subjected to similarly rigorous comparisons. Moreover, the predictability of fruit resources through time has received little attention, and few data appear to exist to address this basic question [7, 10–12].

Long-term phenological studies, that monitor flowering, fruiting, and the flushing and shedding of leaves, are necessary to evaluate the predictability of resources through time. Multi-year monitoring is an important aspect of these studies as inter-annual variation can be high. To fully assess resource availability and predictability it is important for long-term phenology studies to span multiple cycles of climatic variation. In Madagascar, the intensity of phenological monitoring has been variable, with some ecosystems receiving more attention than others. Long-term monitoring (21 years) is ongoing at one site, Beza Mahafaly [13], in the southwest, complementing additional shorter studies in the west (e.g., [14]); and a three-year phenology study monitored the littoral forests of St. Luce in the southeast [15]. In Madagascar’s eastern wet forests—which harbor much of the island’s biodiversity [1]–the duration of publicly available phenology studies is between one and two years [15], and the majority are from a single site, Ranomafana National Park [7, 10–12]. One of the most widely cited and influential of these wet forest phenology studies [11, 12] monitored 104 trees belonging to 26 species that contributed to the diet of brown lemurs (*Eulemur fulvus* and *Eulemur rubriventer*) in Ranomafana, and it documented a five-month period during which there was a scarcity of fruits. Based on these observations, Overdorff [11, 12] and others [6, 7] concluded that fruiting phenology is unpredictable and resources are unreliable in Malagasy forests, supporting the EFH. These wet forest studies have been influential in subsequent research in Madagascar, despite their limited duration, small sample sizes and, importantly, the lack of direct comparisons to tropical wet forests elsewhere.

In order to further evaluate the hypothesis that fruiting resource availability is peculiarly unpredictable in Madagascar, we carried out a comparison of two tropical wet forests—the Réserve Naturelle Intégrale Betampona in Madagascar, and Kibale National Park in Uganda. Specifically, at Betampona we monitored fruiting phenology within the home range of a group of Madagascar’s largest extant frugivore, the black-and-white ruffed lemur (*Varecia variegata*) [16, 17]; and at Kibale, we monitored fruiting phenology among trees that provided food resources to frugivores. We also compared 15-year temperature records from both Betampona and Kibale to assess the unpredictability of climate through time and its possible relation to resource availability. While our phenology study at Betampona has a much larger sample size than earlier studies, we were only able to monitor the site for two years—a timespan comparable to previous studies in the region. Nonetheless, this study provides an analytical framework for future work, highlights a potentially significant difference in total fruit availability between the two sites, and raises doubts about the assumption that the unpredictability of fruit resources explains some unusual aspects of the Malagasy biota.

## Methods

### Site descriptions and phenological data collection

The Réserve Naturelle Intégrale Betampona (Betampona) is a mid-elevation (276–650 m) moist forest in northeastern Madagascar at 17’15”-17’55”°S and 49’12”-49’15”°E. The reserve covers 2,228 ha, receives an average annual rainfall of 2000 mm, and has a mean annual temperature between 21° and 24°C [18]. There are no totally dry months, and the heaviest rain occurs between December and April. The terrain is steep and undulating with numerous ridges. Roughly 70% of the reserve is primary forest, and the remainder has been exposed to different levels of selective logging and other anthropogenic disturbances; it is surrounded by subsistence farming communities.

Kibale National Park sits near the foothills of the Rwenzori Mountains in southwestern Uganda covering 79,500 hectares at 0°13’-0°41’N and 30°19’-30°32’E [19, 20]. The area is an evergreen, mid-altitude (1,500 m) forest near Makerere University Biological Field Station. Rainfall varies bimodally during the year, with an annual average of 1,680 mm between 1990–2015 (CAC unpublished data; [21]). The mean annual temperature ranges between 19.1 and 21.4 Co. The phenological monitoring reported here focused on a site (K-30 forestry compartment) that has never been commercially harvested.

To quantify the resources that a frugivore might encounter at Betampona over the course of a year, four transects were established; three of them 20 by 200 meters and one 20 by 50 meters, for a total of 1.3 hectares. All work carried out in Betampona was approved by the Madagascar National Parks Service (http://www.parcs-madagascar.com/). These transects all occurred within the known home range of one *Varecia variegata* (black-and-white ruffed lemur) community. This is the largest extant frugivore in Madagascar, and also the most obligately frugivorous species of lemur on the island [22–24]. Within each transect, all trees with a diameter at breast height (DBH) of 10 cm or larger that were documented as contributing to *V*. *variegata* diet were tagged for phenological monitoring [18, 22]. By focusing on *V*. *variegata* food trees, we ensured that we monitored a community’s resource availability rather than fruiting phenology irrelevant to this key frugivore. For each tagged tree, height, DBH, altitude, and a georeferenced locality were recorded (S1 Table). A total of 869 individuals from an estimated 27 species in 23 genera, with an average of 33 individuals per species (ranging from 1 to 148 individuals per species), were monitored between September 2013 and September 2015 (S1 Table).

At Kibale, phenological data were collected over 185 months (May 1998 to September 2014; Chapman and colleagues unpublished data). Monthly phenological patterns were recorded using a trail system covering a total of 5.2 hectares that monitored ~326 individuals from 43 species (all of them food trees for frugivores at Kibale), and an average of 8 individuals per species (with a range of 1–13 individuals). The number of individuals varied slightly over time as trees occasionally died; replacement trees of the same species and height were added to the monitoring regime within two months.

At both sites phenological monitoring was conducted using the same methodology. The crown of each tree was visually examined on a monthly basis to determine the presence of different leaf stages (i.e., leaf buds, young leaves, and mature leaves), flowers, and unripe and ripe fruit. The relative abundance of fruit was evaluated on a scale of 0–4, which proved to be consistent at Kibale between observers [20, 25, 26]. To mitigate possible issues of observer bias, the same observers evaluated fruit abundance for the duration of the study period at Betampona. Rainfall, humidity, and temperature were recorded daily within 2 km (Betampona) and 1 km (Kibale) of the transects. Climate data were collected for each site using a standard weather station and a large circular rain gauge; from these data we obtained mean monthly rainfall and mean monthly maximum and minimum temperatures. At Kibale, solar radiation was also estimated based on data acquired from the Satellite Application Facility on Climate Monitoring (CM SAF) [27].

### Statistical analyses

Fruiting phenology from the long-term Kibale dataset were divided into 2-year intervals of consecutive months that corresponded to the monitoring period at Betampona, for a total of 7 intervals. Total fruit availability for each site was calculated as the relative proportion of fruiting trees to all monitored trees (the activity index), following [28]. To compare the predictability of fruit availability between the two sites we employed three statistical strategies. (1) To identify possible environmental correlates of fruiting patterns, we used linear regressions. (2) To quantify resource predictability, we used Colwell’s [29] index of predictability (*P*) of periodic phenomena in phenological and climatic patterns. As temperature was the most significant climatic predictor of Betampona’s fruiting phenology, for both sites we calculated the *P* of fruiting phenology and temperature. (3) To evaluate fluctuations in total fruit availability, we quantified the frequency and duration of months with low fruit availability (defined below). All statistical analyses were performed with the statistical programming platform R v 3.1.2 [30].

Linear regression was performed in the *‘stats’* package [30] to test for climatological correlates of fruit availability. To detect and control for collinearity between predictor variables, which can cause instability in parameter estimation [31], we used a stepwise variance inflation factor (VIF) calculation to eliminate the variables that demonstrated the most collinearity [32]. Stepwise VIF calculates the VIF for all pairs of predictor variables and excludes those with values exceeding a threshold of 5 and retaining all others [31]. To account for a lag time between potential climatic effects on phenology, we also calculated the lag time between fruiting patterns and VIF-selected climatological variables, following [33], and incorporated those lags into our analyses. To identify significant climatic predictors of fruiting phenology, we used our VIF-selected variables in a stepwise regression with forward selection and backward elimination based on AIC scores. These VIF-selected climatic variables were: for both sites, (1) total monthly rainfall, (2) average daily humidity, (3) average temperature; and for Kibale only, (4) average solar radiation. Residuals of the final models were plotted and confirmed as normally distributed.

To quantify the predictability of total fruit availability and temperature, we used Colwell’s index [29]. In Colwell's index [29], *P* (predictability) is the sum of Constancy (*C)* and Contingency (*M)*. *C* describes the extent to which the variable of interest is similar to or equivalent between time points, here the similarity of mean fruit availability or temperature from month to month. *M* calculates the extent to which the variable of interest is similar for equivalent time points between years, here the similarity of mean temperature or fruit availability in a given month from year to year. *P* of fruit availability was calculated for the two years of Betampona data, and for each of the 2-year Kibale intervals, as well as for the entire 15-year Kibale dataset (‘*Hydrostats’* package [34]). For average temperature, a 14-year dataset was available for Betampona, so *P* was calculated for all years at both sites (‘*Hydrostats*’ package [34]). To account for variance, we conducted 100 bootstraps (keeping years constant) of *P* for both temperature and fruit availability.

Finally, the frequency and duration of periods of resource scarcity were evaluated, assessing when monthly total fruit availability fell below either 10% or 25% of maximum observed fruiting. These levels were selected based on recorded percent fruit available in previous Malagasy phenology studies during timespans highlighted in the literature as prolonged periods of scarcity [6, 7, 11].

## Results

**Fig 1:**
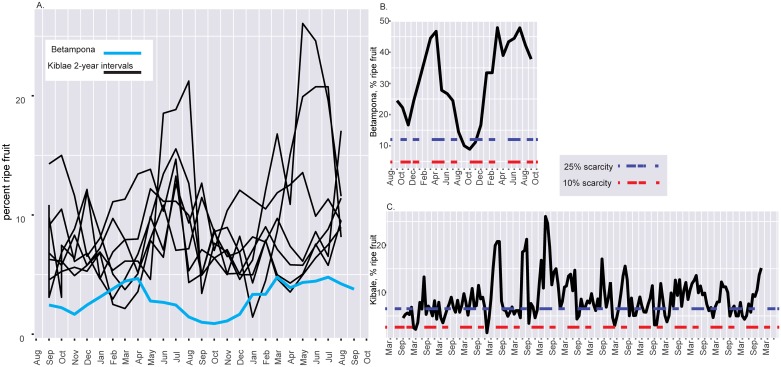
Fruit availability and scarcity at Betampona and Kibale. **(A)** Total fruit availability at each site. Kibale phenology data are presented in 2-year intervals with black lines; Betampona phenology data are shown in light blue. Periods of fruit scarcity at Betampona **(B)** and Kibale **(C)**; dark blue lines indicate 25% of the maximum availability, red lines indicate 10%.

Although Betampona had a consistently lower relative number of fruiting trees than Kibale, the tempo of fruit availability at Betampona, and over any 2-year interval at Kibale, followed roughly the same general pattern (Fig 1). Linear regressions on a two-month (Betampona) and ten-month (Kibale) time lag showed that fruiting at both sites can be predicted by temperature at Betampona, or by temperature in combination with solar radiation at Kibale, rather than by rainfall or humidity (Table 1). In the best-fit model for Betampona, temperature explains 34% of the variation in fruit availability (Table 1B). For Kibale’s best-fit model, solar radiation and average temperature combined explains 14% of the variation in fruit availability (Table 1D).

**Table 1:** Stepwise linear regressions to test for climatological correlates of fruit availability in Betampona, Madagascar (A, B) and Kibale, Uganda (C,D).

A. Betampona, Model 1^1^	Coefficient	Standard error	T-value	P-value
Total rainfall	1.39*10^−5^	1.71*10^−5^	0.816	0.4238
Average humidity	-2.59*10^−4^	1.13*10^−3^	-0.230	0.8205
Average temperature	0.0025	1.30*10^−3^	1.958	0.0643
**B. Betampona, Model 2**^2^				
Average temperature	2.97*10^−3^	8.817*10^−4^	3.372	0.0028
**C. Kibale, Model 3**^3^				
Average temperature	7.31*10^−3^	3.80*10^−3^	1.950	0.0530
Average rain	-7.70*10^−4^	1.00 *10^−2^	-0.750	0.4600
Average solar radiation	5.6*10^−4^	2.00 *10^−4^	2.950	0.004
**D. Kibale, Model 4**^4^				
Average temperature	7.491*10^−3^	3.74*10^−3^	2.002	0.0470
Average solar radiation	6.082*10^−4^	7.53*10^−4^	3.469	0.0007

^1^Stepwise regression: direction = both; R^2^ = 0.37, F = 3.85, on 3 and 20 df, p-value 0.025

^2^Significant predictor variable from Model 1: R^2^ = 0.34, F = 11.37, on 1 and 22 df, p-value 0.0027)

^3^Stepwise regression (direction = both, R^2^ = 0.14, F = 8.33, on 3 and 154 df, p-value 3.59E-05)

^4^ Significant predictor variables from Model 3 (R^2^ = 0.14, F = 12.26, on 2 and 155 df, p-value 1.142E-05)

**Fig 2:**
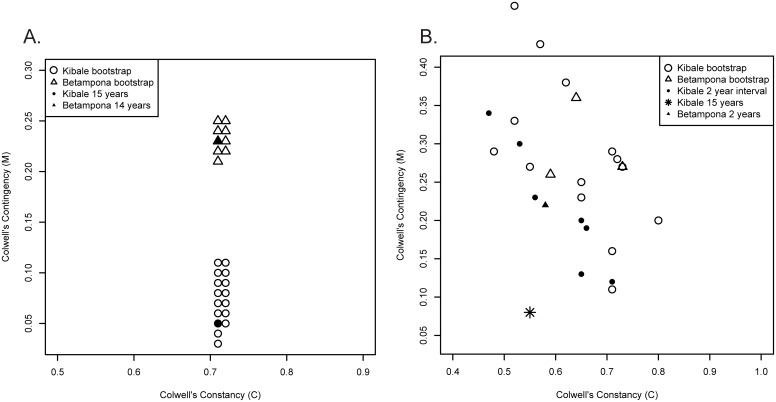
A comparison of Colwell’s index of predictability *(P)* of temperature and fruit availability at Kibale and Betampona. **(A)** Colwell’s constancy (*C)* versus contingency (*M)* for 15 years of temperature data at Kibale (filled circle) and Betampona (filled triangle); both have been bootstrapped 100 times (open triangles and circles). **(B)** Colwell’s constancy (*C)* versus contingency (*M)* for 15 years and for two-year intervals of total fruit availability at Kibale (star and filled circles, respectively), and 2-years of fruit availability at Betampona (filled triangle); both have been bootstrapped 100 times (open triangles and circles).

Fourteen years of temperature data at Betampona were more seasonal (more variation month to month), but also more predictable overall, than 15 years of data at Kibale; this is primarily a function of higher *M*, i.e., lower inter-annual variability (Betampona: Colwell’s *P* = 0.94, *M* = 0.23; Kibale: *P =* 0.77, *M* = 0.05) (Fig 2). Analyses of fruit availability showed a similar pattern: while total fruit availability was generally higher at Kibale than at Betampona, Betampona proved more predictable than the 15-year Kibale dataset, again due to lower inter-annual variability (Betampona, *P =* 0.80, *M =* 0.22; Kibale *P* = 0.64, *M =* 0.08; Fig 2B; Table 2). However, when Colwell’s index was used to compare total fruit availability at Betampona with Kibale’s two-year intervals, both sites fell within the same range (Fig 2B, Table 2).

**Table 2:** Colwell’s index of predictability for fruit availability: comparing two year intervals from Kibale, Uganda with two years of data from Betampona, Madagascar.

Site	Interval	Colwell's P	Colwell's C	Colwell's M
Kibale	1999–2001	0.78	0.56	0.23
Kibale	2001–2003	0.83	0.53	0.3
Kibale	2003–2005	0.81	0.47	0.34
Kibale	2005–2007	0.78	0.65	0.13
Kibale	2007–2009	0.85	0.65	0.2
Kibale	2009–2011	0.85	0.66	0.19
Kibale	2011–2013	0.83	0.71	0.12
Betampona	2013–2015	0.8	0.58	0.22

Over a two-year period Betampona experienced no months when fruiting fell below 10% of the maximum observed amount of available fruit, while over a 15-year time frame Kibale experienced 7 such months (Fig 1C). When scarcity is defined as less than 25% of the maximum observed amount of available fruit, Betampona experienced three consecutive months of scarcity (12.5% of the total study period) (Fig 1B). Over the entire 15-year study period Kibale experienced 71 months of at least 25% scarcity (39.4% of the total study period) (Fig 1C); while these months were spread over the 15-year monitoring period, there were nine times where periods of scarcity constituted three or more consecutive months. This amounts to a period of scarcity at Kibale roughly every 1.5 years (Fig 1C).

## Discussion

Based on an assessment of the two-year interval data from each site, we found no significant difference in patterns of resource predictability between Betampona and Kibale (Fig 1), which raises doubt about the Energy Frugality Hypothesis (EFH). While fruit resource availability for two years of monitoring at Betampona (*P* = 0.80) appears to be more predictable than over 15 years at Kibale (*P* = 0.64), this difference could disappear with a longer monitoring period at Betampona. Indeed, in comparing two-year intervals at Kibale to the monitoring period at Betampona, fruiting predictability at Betampona and Kibale fall within the same range (Fig 2, Table 2). In any case, during the two years examined at each site, Betampona showed no long periods of fruit scarcity comparable to those observed by Overdorff [11, 12] at Ranomafana. This may reflect our larger sample size at Betampona (863 versus 124 monitored trees) or possibly site-specific phenological differences.

Rather than predictability, the primary difference that we observe between the two sites is the notably lower relative number of fruiting trees at Betampona. Total fruit availability sometimes overlapped between the sites, but only during periods of relative scarcity at Kibale (Fig 1). While the general dearth of resources at Betampona could be an artifact of our short-term monitoring, it seems possible that generally low levels of fruit (rather than lower predictability as per the EFH) could account for Madagascar’s paucity of frugivorous lemurs. It is important to note, however, that, while we did not conduct species-level analyses, the monitoring at Kibale included more species than at Betampona (43 versus 27 species, respectively), and it is possible that this alone accounts for the difference we found in resource abundance. We also note that it is not clear whether the relatively low levels of fruit observed at Betampona are abnormal with respect to other tropical forests. A survey of total fruit availability from other long-term tropical phenology studies finds that the number of fruiting trees at any one time can vary between 3% and 20% of the total monitored population [28, 33, 35, 36]. Both Betampona and Kibale fall within this range of variation, though at different ends of the distribution.

Correlations between resource availability and unpredictable climatic variables could favor the hypothesis that it is a hypervariable environment that results in unreliable fruit resources. Our analyses indicate that temperature is the strongest correlate of fruiting phenology in the moist forests of Madagascar, predicting roughly one third of resource availability (Table 1B). This is consistent with most comparable phenological studies across species and latitudes [37]. While we have only two years of fruiting phenology data at Betampona, temperature predicted roughly one third of resource availability, and the long-term temperature record from the site allows us to assess predictability using temperature as a proxy. Furthermore, as most hypotheses about frugivores on Madagascar rely on climatic predictability, understanding the long-term variability and predictability in temperature is important in its own right. Over a 14-year period, we found that temperature at Betampona (*P =* 0.94) was more predictable than at Kibale (*P =* 0.77), largely due to higher inter-annual predictability (Fig 2). This result suggests that temperature in Madagascar, unlike rainfall [2], may be no less predictable than in other tropical areas.

Typically, fleshy-fruited tree species reliant upon animal dispersal constitute 60–90% of rainforest biome tree floras [38], and Madagascar is no exception [39]. Given its ‘normal’ proportion of fleshy-fruited plant species, there appears to be a mismatch between the low diversity of frugivorous animals (primates and birds alike) in Madagascar and the abundance of plants that presumably rely upon them. Contrary to the EFH [6], our results suggest that predictability in Malagasy moist forest fruiting phenology may not differ significantly from a mainland African site. If Malagasy resources are no more unreliable than mainland counterparts, hypotheses that assume more unpredictable resources will need to be re-evaluated.

The fact that Madagascar is depauperate in frugivores compared to other tropical areas [40, 41] could be explained by other evolutionary factors aside from environmental unpredictability. Madagascar has been isolated for 88 million years, and during that time, very few vertebrate lineages have colonized the island [2, 42]. Over such a long time period, we might expect broad convergence in functional traits and ecological assemblages, regardless of the identity of the colonizers [43–45]. However, it is possible that the sweepstakes dispersal necessary to colonize isolated landmasses could result in the arrival and persistence of lineages that are ill-suited to converge on a particular distribution of functional traits. It is also important to appreciate that megafaunal extinctions in Madagascar over the past three thousand years probably resulted in a dramatic reduction of frugivorous species, including elephant birds (*Aepyornithidae* spp. [46]) and many lemur species [47, 48]. It is, however, difficult to accurately estimate the historical size and composition of frugivores in Madagascar owing to the lack of a Cenozoic fossil record.

Our analyses address the role of environmental variability in shaping the curious biodiversity of Madagascar, and highlight the importance of critically testing assumptions about the origins of that biodiversity. While our study does not find greater unpredictability in fruiting resources in Betampona as compared to Kibale, we still cannot rule out the hypothesis as it relates more broadly to Malagasy wet forests; the relatively short duration of observations at any Malagasy site is inadequate to address the issue of unpredictability definitively [20, 28]. Our data do, however, raise the alternative possibility that the lower abundance of fruiting trees at any one time may have limited frugivore diversity. Clearly, longer-term phenological studies are needed across Madagascar, especially in the wet forests, as well as systematic comparisons among tropical forests more broadly. In the meantime, our findings, in combination with those from long-term phenological studies elsewhere (e.g., Ivory Coast [49] and Costa Rica [50]), will help to clarify the role of environmental variability, convergence, and ecological filtering in structuring tropical plant and frugivore communities.

## Supporting Information

S1 Table Betampona phenological monitoring information. Data on the common name, identification, locality, height, diameter at breast height (DBH), and elevation for every tree included in phenological monitoring at Betampona. Species were identified by matching a Betampona-specific common name to an index of species developed by botanist Bernard Iambana in collaboration with the Missouri Botanical Garden.
(CSV)
